# SARS-CoV-2 Delta Variant is Recognized Through GRP78 Host-Cell Surface Receptor, In Silico Perspective

**DOI:** 10.1007/s10989-022-10450-w

**Published:** 2022-08-22

**Authors:** Abdo A. Elfiky, Ibrahim M. Ibrahim, Alaa M. Elgohary

**Affiliations:** Biophysics Department, Faculty of Science, Cairo University, Giza, Egypt

**Keywords:** SARS-CoV-2 new variants, B.1.617, Computational biophysics, GRP78, Spike RBD

## Abstract

Different SARS-CoV-2 new variants emerged and spread during the past few months, sparking infections and death counts. The new variant B.1.617 (delta variant) sparked in India in the past few months, causing the highest records. The B.1.617 variant of SARS-CoV-2 has the double mutations E484Q and L452R on its spike Receptor Binding Domain (RBD). The first mutation is like the reported South African and the Brazilian variants (501.V2 and B.1.1.248). This mutation lies in the region C480-C488, which we predicted before to be recognized by the host-cell receptor; Glucose Regulated Protein 78 (GRP78). In the current study, we test the binding affinity of the host-cell receptor GRP78 to the delta variant spike RBD using molecular docking and molecular dynamics simulations of up to 100 ns. Additionally, the ACE2-RBD is tested by protein–protein docking. The results reveal equal average binding affinities of the GRP78 against wildtype and delta variant spikes. This supports our previous predictions of the contribution of GRP78 in SARS-CoV-2 spike recognition as an auxiliary route for entry.

## Introduction

According to the john-Hopkins COVID-19 counter, India reported the highest daily new record worldwide in the number of infections on May 6, 2021. This spark in the highly contagious virus was attributed to the new variant B.1.617 (delta strain). This variant has double mutations, E484Q and L452R, that are suggested to be crucial for viral recognition because it is found in the RBD of the viral spike. The mutant at the 484 position of the spike was reported before in other variants of SARS-CoV-2 such as B.1.1.248 (beta variant) and 501.V2 (gamma variant), in which it was E484K (Ibrahim et al. 2021). In addition, some cities in India were suffering from limited hospital beds, medicines, and oxygen supplies, leading to a death count surge last year (Explainer: What we know about the Indian variant as coronavirus sweeps South Asia; The effects of virus variants on COVID-19 vaccines: WHO 2021).

Fear is now facing the world due to the massive spread of the delta variant (Explainer: What we know about the Indian variant as coronavirus sweeps South Asia). Studying the mutations that emerged in the spike RBD is essential due to its involvement in vaccine recognition (The effects of virus variants on COVID-19 vaccines: WHO 2021). In previous studies, we reported the efficiency dependence of the SARS-CoV-2 variant on its recognition behavior either by the primary recognizing receptor Angiotensin-Converting Enzyme 2 (ACE2) and the cell-surface-GRP78 (CS-GRP78) (Elfiky and Ibrahim 2021a). Additionally, we reported the mutation at position 488 of the spike would affect its binding to ACE2 by breaking a salt bridge (E484-K31) found in the wildtype SARS-CoV-2 spike (Ibrahim et al. 2021).

Previous prediction studies reported the incorporation of the cell-surface receptor (CS-GRP78) in SARS-CoV-2 recognition and possibly facilitating its internalization into the human alveolar cells (Ibrahim et al. 2020; Elfiky 2020a). The predicted recognition site on the spike of SARS-CoV-2 lies in its RBD (C480–C488). This binding was also indicated for other viruses, including Zika, Ebola, Human papillomavirus, and the MERS-CoV (Elfiky 2020b, c; Elfiky and Ibrahim 2021b; Chu et al. 2018). Additionally, GRP78 was suggested as a possible link between COVID-19 and Mucormycosis (Elgohary et al. 2021). Recently an experimental study using Vero E6-ACE2 cells was conducted by Carlos et al*.* that confirmed the association of GRP78 with both SARS-CoV-2 and its primary receptor ACE2 (Carlos et al. 1978). They concluded that the spike protein's receptor-binding domain β (SBD β) is the docking platform for GRP78. Furthermore, they reported that GRP78 is important for the cell-surface localization of ACE2. Additionally, the humanized monoclonal antibody (hMAb159) reduced SARS-CoV-2 entry through the spike by decreasing Cs-GRP78 and Cs-ACE2, thus inhibiting SARS-CoV-2 infectivity in vitro (Carlos et al. 1978).

Computational predictions proved their crucial role in COVID-19 fighting (Mahmud et al. 2021; Sonousi et al. 2021; Gyebi et al. 2021; Wang 2020). In the current study, molecular dynamics simulation for the spike RBD of the delta variant was performed, followed by protein–protein docking to test the efficacy of the binding of the spike to both human cell-surface receptors GRP78 and ACE2.

## Materials and Methods

Protein Data Bank (PDB) database was used to download the solved structures of spike receptor binding domain (RBD) (PDB ID: 6M17 chain E), ACE2 (PDB ID: 6M17 chain B), and GRP78 (PDB ID: 5E84, chain A). Two point mutations (E484Q and L452R) were administered in the spike RBD using the CHARMM-GUI webserver (Jo et al. 2008, 2014). RBD, ACE2, and GRP78 structures were prepared for docking using HADDOCK V2.4 webserver by removing unnecessary molecules such as water and other ligands (except for the oligosaccharides), while missing hydrogen atoms were added. Active sites for each protein were retrieved from literature, for GRP78: T428, V429, V432, T434, F451, S452, V457, and I459 (Yang et al. 2015), while for spike RBD: C480-C488 (against GRP78) and K417, Y453, Q474, F486, Q498, T500, and N501Y (against ACE2) and for ACE2: Q24, D30, H34, Y41, Q42, M82, K353, and R357 (Yan et al. 2020). The easy interface of HADDOCK V2.4 was used, and the remaining settings were set as default.

NAMD V2.13 was utilized to perform Molecular Dynamic Simulation (MDS). CHARMM-GUI web server was used to prepare the RBD and GRP78-RBD complex (produced from HADDOCK) necessary files for MDS (Jo et al. 2008; Phillips et al. 2005; Brooks et al. 2009; Lee et al. 2016). Both systems were solvated in the TIP3P water model, salt concentration was set to 0.154 M NaCl, and the temperature was set to 310 K. Time step was set to 2 fs, and the systems were minimized for 20,000, and 10,000 steps for RBD and GRP78-RBD complex, respectively. This is followed by an equilibration run for one ns in a constant number of atoms, constant pressure, and constant temperature (NPT ensemble) for both systems. The pressure was maintained at 1 atm using a Langevin piston, while the temperature was maintained at 310 K using Langevin dynamics. The systems were allowed to explore their conformational spaces for 100 ns in a constant number of atoms, constant volume, and constant temperature (NVT ensemble) for the production runs. After completing the MDS for the RBD, the equilibrated trajectories were clustered using TTClust V 4.8.3 python library (Tubiana et al. 2018). The number of clusters was determined automatically using the elbow method implemented in the TTClust library. Two clusters were obtained, and for each cluster, a representative frame was selected by the library. HADDOCK V 2.4 was used to dock each representative frame with the GRP78 solved structure using the previously mentioned active sites. Protein–Ligand Interaction Profiler (PLIP) was used to detect the number and types of interaction between the RBD and GRP78 for each S RBD-GRP78 complex produced (Salentin et al. 2015). The same protocol was pursued for S RBD (WT and delta)-ACE2 complex to test its binding affinities.

## Results and Discussion

**Fig. 1:**
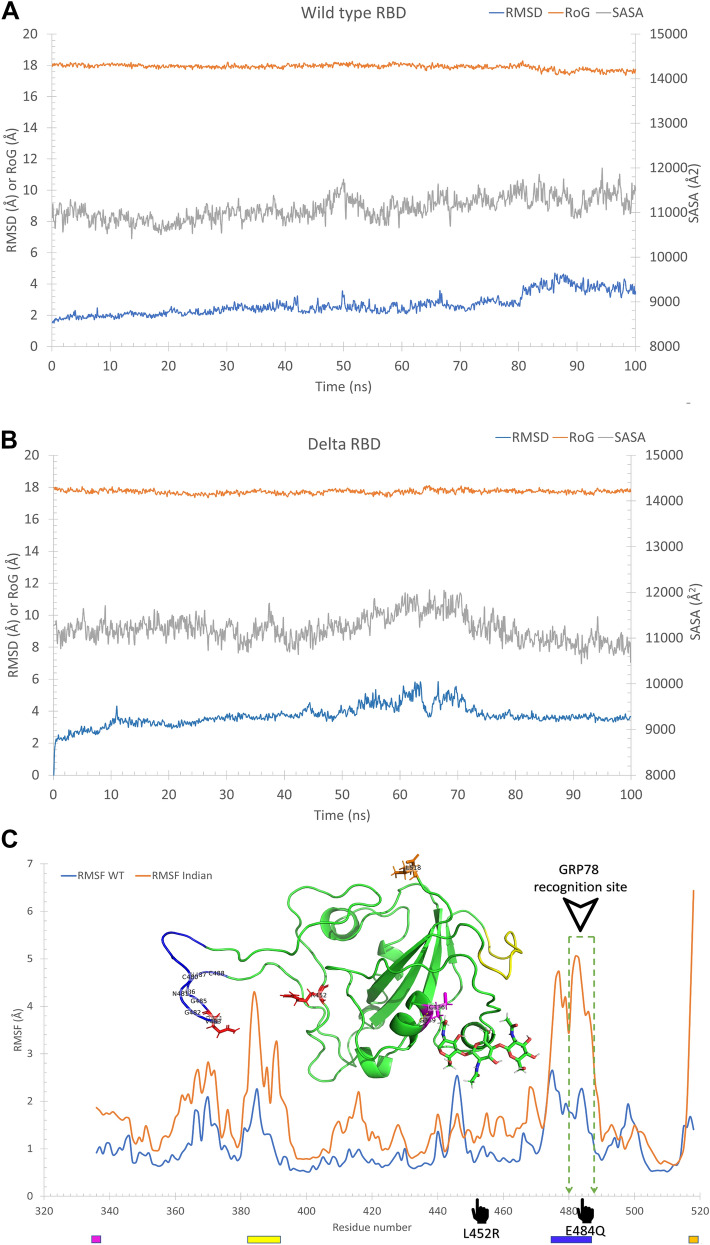
Molecular dynamics simulation of the SARS-CoV-2 spike RBD wild type (**A**) and delta variant (**B**). The Root Mean Square Deviation (RMSD) (blue line), Radius of Gyration (RoG) (orange line), and Surface Accessible Surface Area (SASA) (gray line) versus the simulation time. (**C**) the per-residue Root Mean Square Fluctuation (RMSF) for the WT (blue line) and Indian (delta) (orange line) variants of SARS-CoV-2 spike RBD. The highly fluctuating regions are marked by the colored rectangles on the RMSF curves and colored cartoons in the structure. The mutations L452R and E484Q are marked on the RMSF curve and by red sticks on the structure

The mutated isoform of the SARS-CoV-2 spike (E484Q and L452R) is built and optimized, then subjected to a 100 ns MDS production run alongside the wildtype RBD to prepare the structures for the docking study. The dynamics are performed to explore the possible conformational space of the mutated spike prior to the docking study. The Root Mean Square Deviation (RMSD) in Å, the Radius of Gyration (RoG) in Å, and the Surface Accessible Surface Area (SASA) in Å^2^ are plotted in Fig. 1A and B. As reflected from the plots, the systems were equilibrated (RMSD is flattened) after the first 10 ns of the simulation, with an average value for the RMSD (blue line) of 2.66 ± 0.68 and 3.82 ± 0.54 Å for the wild type and delta RBD, respectively. The systems are equilibrated and stable, as reflected in the values of the RoG (orange line) and SASA (gray line). The average RoG is 17.9 ± 0.16 Å and 17.7 ± 0.12 Å, while SASA has average values of 11,092 ± 260 Å^2^ and 11,207 ± 295 Å^2^ for the wildtype and delta RBD, respectively.

After the MDS, TTClust is utilized to cluster the trajectories using the elbow methods, and we come up with five clusters representing the 1000 trajectories during the 100 ns MDS. We select a representative conformation from each cluster to test the GRP78 binding using HADDOCK 2.4. Tables 1 and 2 summarize the interactions established for each cluster representative docked with GRP78 along with the HADDOCK scores. The mutated residues R452 and Q484 are shown in red color in Table 2. The average HADDOCK score for the delta mutant isoform of the spike RBD is − 72.58 ± 9.5. This score is almost the same as the wildtype (WT) RBD docking score against GRP78 (− 74.3 ± 0.9) (Elfiky and Ibrahim 2021a). The two mutated residues (R452 and Q484) have an impact on the binding of the spike to GRP78, especially Q484. In the five different conformations, Q484 contributed at least one H-bond to the GRP78 RBDβ residues in four conformations. Bold residues in Tables 1 and 2 represent the active residues from both GRP78 and spike selected by HADDOCK to be flexible during searching for the best binding mode. In delta RBD, the most-reported residues from the spike to form H-bonds are N481 (8), S477 (4), and N487 (4), while the residues that form both H-bonds and hydrophobic interactions are F486 (10), Q484 (8), V483 (6), and Y489 (4). On the other hand, the wildtype RBD shows a slightly higher average number of H-bonds (8 ± 1.2) compared to the delta RBD (6.6 ± 1.6) but a slightly lower average number of hydrophobic contacts (3.3 ± 1.3) compared to the delta RBD (5.0 ± 1.1). The most reported residues that form interactions with GRP78 in the case of the wildtype RBD are T428 (5), V429 (5), G430 (4), T434 (4), Q449 (4), and T458 (4).

**Table 1:** The interactions established between GRP78 and the four different conformations of the wild type SARS-CoV-2 spike RBD

Cluster number	HADDOCK score	Number of hydrogen bonds	GRP78 amino acids	RBD amino acids	Number of hydrophobic interactions	GRP78 amino acids	RBD amino acids	Number of salt bridges	GRP78 amino acids	RBD amino acids	Number of π-stacking	GRP78 amino acids	RBD amino acids
1	− 60.0 ± 2.8	6	**T428(2)**, **V429**, **T434,** and Q449(2)	**N481(2)**, **F486**, and S477(3)	4	I426, **T428**, and **V429(2)**	**F486**, T478, **N481**, and **V483**				1	**F451**	**F486**
2	− 81.6 ± 7.5	8	G430(2), **S452**, T456, T458(2), I483, and A486	**N481(2)**, **N487**, **E484(3)**, Q493, and Y453	4	I426, **T428**, **T434,** and P485	**F486(3)** and Q493				1	**F451**	**F486**
3	− 73.6 ± 3.6	9	E427, **T428**, G430(2), G454, T456, T458(2), and G489	T478, **N481**, **F486**, **N487**, E471, Q474(2), K458, and T470	1	**V429**	**F486**						
4	− 70.2 ± 3.9	9	E347(3), **V429**, **T434(2)**, K435, and Q449(2)	Y449(2), N448, **N487**, **E484(3)**, **V483**, and **N481**	4	I426, **V432(2)**, and **F451**	**F486**, Y489(2), and **F486**	1	K435	**E484**	1	**F451**	**F486**

The underlined are the mutated residues

**Table 2:** The interactions established between GRP78 and the five different conformations of the mutated SARS-CoV-2 spike RBD (E484Q and L452R)

Cluster number	HADDOCK score	Number of Hydrogen bonds	RBD amino acids	GRP78 amino acids	Number of hydrophobic interactions	RBD amino acids	GRP78 amino acids	Number of salt bridge	RBD amino acids	GRP78 amino acids
1	− 83.2 ± 1.4	9	K444, V445, R452, **N481(2)**, ** Q484**, **F486**, **N487,** and **Y489**	**V429(2)**, **T434**, K447, Q449, **S452**, V490, G515, and N516	5	**V483**, **Q484**, **F486(2)**, and F490	L436, K447, **F451**, V453, and V490			
2	− 55.5 ± 4.5	6	S477, T478, **N481**(2), ** Q484**, and **N487**	**T428**, **T434**, I450(2), **S452**, and G454	6	P479, ** Q484(2)**, **F486,** and **Y489(2)**	**V429(2)**, **V432(2)**, **F451**, and **V457**			
3	− 74.1 ± 3.3	5	T345, R346, K444, **N481**, and ** Q484**	W103, N104, E121, **S452**, and T458	5	I472, **V483(3)**, and **F486**	**I426**, E427, **V429**, V453, and K460			
4	− 79.3 ± 1.9	8	S477(3), **N481(2)**, ** Q484**, and **N487(2)**	**T428**, **S452(2)**, T456(3), and T458(2)	6	**Q484**, **F486(4)**, and **Y489**	**I426**, **V429(2)**, **F451**, **V457**, and **I459**			
5	− 70.8 ± 7.6	5	**N481**, **V483**, T500, N501, and G502,	E243(3), **S452**, and Q492	3	A478, **V483**, and **F486**	**T434**, **F451**, and V490	1	R408	D350

The underlined are the mutated residues

Bold represent the active site residues (C480-C488)

Figure 1C shows the per-residue Root Mean Square Fluctuations (RMSF) for the wildtype spike (WT) (blue line) and the delta variant spike (orange line) after 100 ns MDS runs. The structure of the spike RBD is shown in green cartoons. For the delta variant, the most fluctuating regions (RMSF < 3 Å) are depicted in different colors in the structure and marked on the RMSF curve as well. Two regions show high fluctuations, the yellow region (P384-F392) and the blue region (Q474–Q488), with RMSF reaching 4.3 and 5 Å, respectively. The C-terminal residue (orange sticks) also shows high RMSF (6.4 Å), while the N-terminal residue C336 (magenta sticks) is stabilized by the formed H-bond (dashed-yellow line) to G339. The dashed-green region marks the GRP78 recognition site (C480-C488) on the RMSF curve. This region fluctuates in the delta variant (RMSF of up to 5.00 Å) compared to the WT RBD (RMSF less than 2.26 Å). The mutation E484Q (red stick) also lies in this region, which may be the reason for the increased flexibility of this blue loop.

**Fig. 2:**
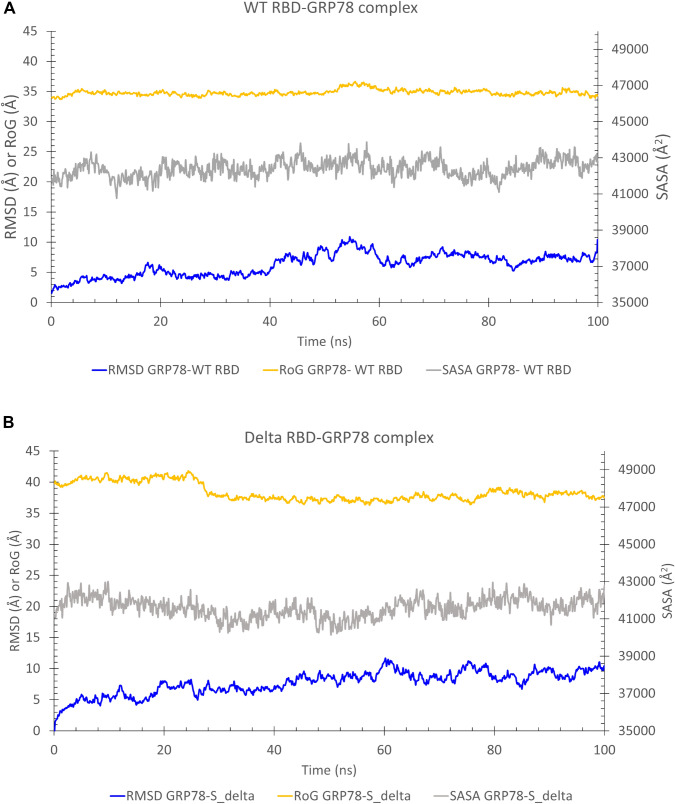

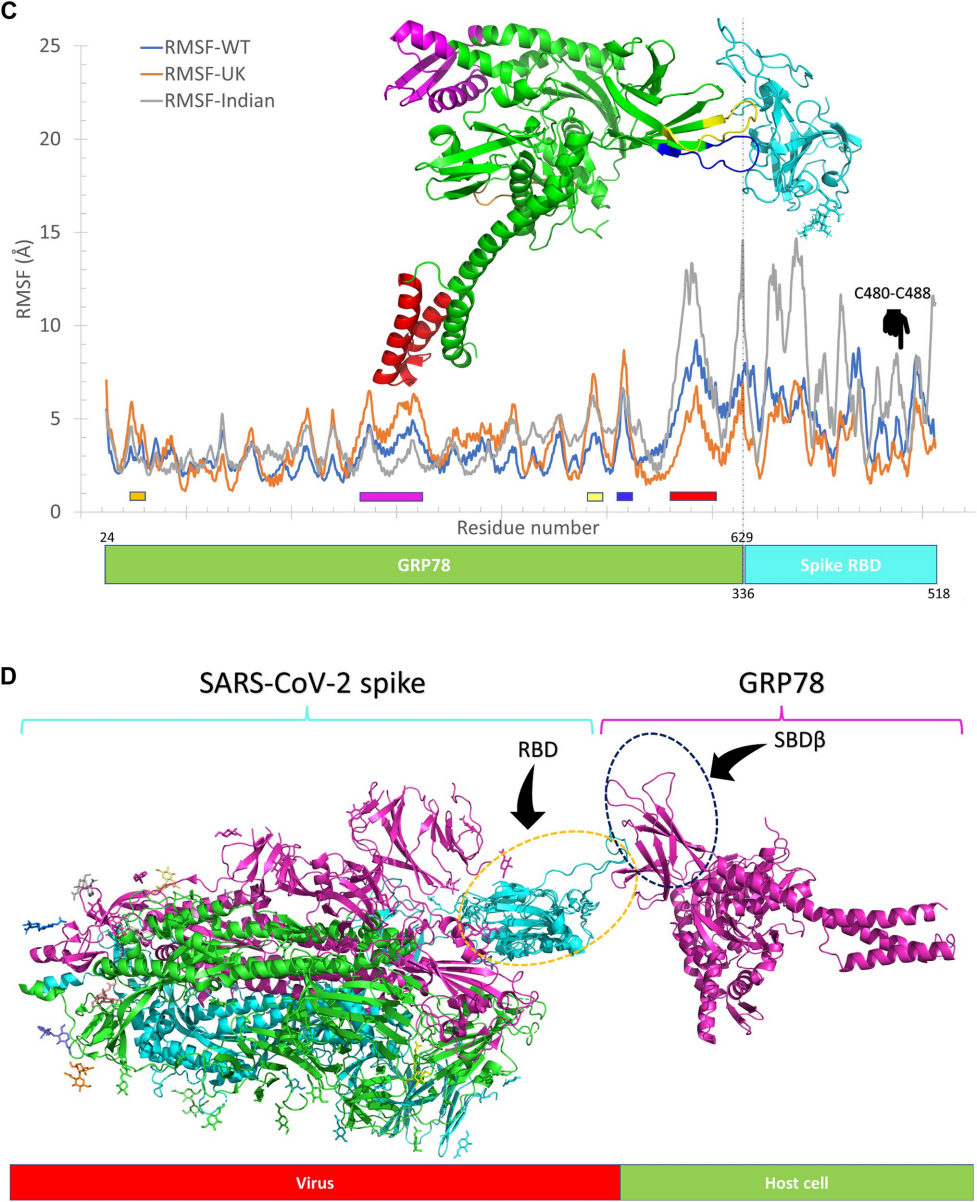
Molecular dynamics simulation of the RBD-GRP78 complexes. **A** and **B** The Root Mean Square Deviation (RMSD) (blue line), Radius of Gyration (RoG) (orange line), and Surface Accessible Surface Area (SASA) (gray line) versus the simulation time for the wild type RBD-GRP78 and delta RBD-GRP78 complexes. **C** The per-residue Root Mean Square Fluctuation (RMSF) for the WT, UK, and Indian (delta) variants of SARS-CoV-2 spike RBD -GRP78 complexes. The highly fluctuating regions are marked by the colored rectangles on the RMSF curves and colored cartoons in the structure. **D** The superposition of the solved structure of SARS-CoV-2 Spike (PDB ID: 6VYB) and the complex of RBD (delta)-GRP78 we modeled

We reported in a previous study on the SARS-CoV-2 spike recognition site by host cell surface GRP78 (Ibrahim et al. 2020). This recognition site lies in the spike's receptor-binding domain (RBD), the same domain that binds human ACE2 (Elfiky 2020a; Elfiky et al. 2021a). New experimental work by Carlos et al*.* supported our prediction. At the same time, the cover artwork of the journal of biological chemistry for the July 2021 issue shows how the recognition occurs between GRP78 and the spike (Carlos et al. 2021). We run MDS for 100 ns for the complex formed between the GRP78 and the delta variant spike RBD in the current study. Figure 2A and B show the RMSD in Å, the RoG in Å, and the SASA in Å^2^ versus time in ns for the wildtype RBD-GRP78 complex (A) and delta RBD-GRP78 complex (B). As reflected from the plots, the systems are equilibrated at the middle of the simulation with an average value for the RMSD (blue line) of 6.3 Å and 9.2 Å for the wild type RBD-GRP78 and delta RBD-GRP78 complexes, respectively. The systems are equilibrated and stable as reflected also from the RoG (orange line) and SASA (gray line) values. The average RoG are 34.8 Å and 38.0 Å, while SASA has average values of 42,407 Å^2^and 42,000 Å^2^ for the wild type RBD-GRP78 and delta RBD-GRP78 complexes, respectively. The values of the RMSD, RoG, and SASA of the complexes (Fig. 2) are larger than that of the spike RBD alone (Fig. 1). This is due to complexity of the system in the case of GRP78-spike RBD compared to RBD alone.

Additionally, the RMSF (in Å) of the GRP78-spike delta RBD complex is depicted in Fig. 2C (gray line) alongside the wildtype (WT) and the beta (UK) strain RBD-GRP78 complexes (blue and orange lines, respectively). Five regions of the GRP78 (green cartoon) are found to be highly flexible in the delta spike RBD-GRP78 complex (RMSF < 5 Å). These include; F45-G58 (orange), F266-K326 (magenta), L480-I494 (yellow), D511-I522 (blue), and L561-S604 (red). These regions are declared on the RMSF curve with colored rectangles and depicted in the structures by colored cartoons. Noticeably, the yellow and blue regions (L480-I494 and D511-I522) are the loops that are involved in the interaction with the RBD of the spike. These two regions are more flexible in the UK and the Indian variants of the RBD-GRP78 complexes (orange and gray curves) compared to the WT RBD-GRP78 complex (blue curve). This increased flexibility may be a reason for the increased susceptibility of the delta RBD to be recognized by different host-cell receptors.

We superimposed the generated model of the RBD (delta) -GRP78 complex with the solved structure of the full-length spike of SARS-CoV-2 (PDB ID: 6VYB) (see Fig. 2D). GRP78 is shown in the magenta cartoon, while the superimposed RBDs are shown in the cyan cartoon. The spike homotrimer is shown in green, cyan, and magenta cartoons. This model represents the recognition of the virus by the host cell surface GRP78 (Ibrahim et al. 2020; Elfiky 2020a; Elfiky et al. 2021a). As reflected from the superposition, the two RBDs (solved structure and the interaction model with GRP78) coincide with each other except for the GRP78 recognition loop (C480–C488), which is missing in the solved structure. This loop is highly flexible and hence missing in the electron density map. The flexibility of this region is the highest in the delta RBD (gray) compared to the wildtype (blue) and UK variant (orange), as shown in the RMSF in Fig. 2C.

On the other hand, the recognition of ACE2 to SARS-CoV-2 Spike RBD delta is tested using the same protocol. Tables 3 and 4 show the detailed interactions established upon docking the ACE2 (PDB ID: 6M17 chain B) against the RBD WT (Table 3) and RBD delta (Table 4) utilizing the HADDOCK 2.4 web server. Bold residues indicate the active residues used to drive the docking in HADDOCK. At least 9 H-bonds and three hydrophobic contacts are established between the two proteins (GRP78 and the WT and delta RBDs), with a salt bridge formed in some conformations. The mutated residues in the delta strain found in the spike RBD (L452R and E484Q) do not contribute to the interaction with ACE2 except in one conformation in which Q484 is involved in H-bond formation, and R452 is involved in a salt bridge. The average docking score for the five conformations is − 106.52 ± 6.9, which is 18.4% higher than the docking score of the WT RBD to ACE2 (Ibrahim et al. 2021). This means that the binding affinity of the ACE2 to the delta variant of SARS-CoV-2 is about 18% lesser than its binding affinity against the WT SARS-CoV-2 spike.

**Table 3:** The established interactions upon docking the wild type RBD into ACE2 (PDB ID: 6M17) using HADDOCK 2.4 web server

Cluster number	HADDOCK score	Number of hydrogen bonds	ACE2 amino acids	RBD amino acids	Number of hydrophobic interactions	ACE2 amino acids	RBD amino acids	Number of salt bridges	ACE2 amino acids	RBD amino acids
C1	− 105.9 ± 3.3	9	**Q24**, **D30,** K31, **H34**, E35, **Y41**, **K353(2)**, and R393	N487, K417, Q493, S494, Q493, **T500**, G502, G496, and Y505	7	**Q24,** T27, **D30**, K31(2), **Y41**, and M82	A475, F456(4), **Q498**, and **F486**	1	K31	E484
C2	− 101.5 ± 1.2	10	**Q24(2)**, **D30**, **H34(2)**, E35(2), D38, **Y41**, and **K353**	S477, N487, K417, **Y453**, G496, Q493(2), **Q498**, **T500**, and G496	3	**Q24**, T27, and K31	A475, and F456(2)	1	K31	E484
C3	− 97.8 ± 3.3	11	**Q24(2)**, **D30,** K31(2), **H34**, D38, **Y41**, Y83(2), and **K353**	N487(2), **K417**, Q493(2), S494, **Q498**, **T500**, Y489(2), and Y505	5	T27, **D30**, L45, M82, and Y83	Y473, F456, **T500**, and **F486(2)**			
C4	− 127.1 ± 11.5	14	**D30(2)**, **H34**, D38(2), **Q42(3),** N49(2), N64, K68, **K353**, and D355	Y489(2), L492, **Y453**, S494, N501(3), R403, G496, Y505, D405, **Y453**, and **K417**	7	**D30**, N33, L45, A46, N64, T92, and P389	Y489(2), Y505(2), **T500**, and **F486(2)**

**Table 4:** The established interactions upon docking the RBD delta into ACE2 (PDB ID: 6M17) using HADDOCK 2.4 web server

Cluster number	Haddock score	Number of H-bonds	RBD amino acids	ACE2 amino acids	Number of hydrophobic interactions	RBD amino acids	ACE2 amino acids	Number of salt bridge	RBD amino acids	ACE2 amino acids
1	− 108.0 ± 3.3	9	G446, **Y453**, Y473, **Q474**, Q493, **Q498**, **T500**, and **N501(2)**	**Q24(2)**, **D30**, K31, E37, **K353(2)**, A386, and R393	7	Y449, L455(2) and **F486(4)**	T27, **D30**, **H34**, E75, T78, and L79(2)	1	R403	**D30**
2	− 107.0 ± 1.7	16	**K417**, Y449, **Y453**, Y473(2), **Q474**, G476, N487, Y489(2), Q493(2), **Q498**, **T500**, **N501**, and Y505	**Q24(2)**, T27, **D30**, K31, **H34(2)**, E35, D38, **Y41**, **Q42**, Q76(2), L79, Y83, and **K353**	5	L455, F456, A475, **F486** and Y489	T27,F28(2), K31, and L79			
3	− 95.6 ± 11.0	14	Y421(2), **Y453**, R454, Y473, S477, N487, Q493(2), S494(2), **Q498**, and **T500(2)**	**Q24(2)**, T27, **D30(3)**, K31, N33, **H34**, D38, N322, **K353(2)**, and M383	5	Y449, **Y453**, A475, and **F486(2)**	T27, **H34,** A386, F555, and R559			
4	− 117.1 ± 1.3	12	**K417**, Y449, L455, *Q484*, G485, N487, Y489, Y495, **Q498**, **T500(2)**, and Y505	E23, **Q24**, K31, **H34(2)**, D38, **Q42**, K74, E75, T78, L79, and **K353**	3	Y489(3)	E75, T78, and L79	1	*R452*	E75
5	− 104.9 ± 15.9	18	Y449, **Y453**, L455, S477(2), T478, N481(2), N487(2), Y489(2), Q493(2), **Q498**, **T500**, and **N501(2)**	T27, **D30**, K31, E35(2), D38, **Y41**, **Q42**, Q76(2), Y83, Q325, E329(2), N330(2), **K353**, and D355	5	L455, A475, **F486**, and Y505(2)	K31, D38, **Y41**, F72, and D355	1	R403	E75

Underline residues represent the π-stacking interactions, while italic residues are the mutated residues of the RBD delta strain (L452R and E484Q)

Bold represent the active site residues (C480-C488)

Conclusively, the binding affinity of the delta strain spike RBD against the host cell receptors ACE2 and GRP78 is reduced in the former but maintained in the latter. This reflects the increased contribution of GRP78 in viral recognition in the delta RBD versus the wildtype RBD. This increased contribution of the GRP78 recognition was reported for the other variants of SARS-CoV-2 compared to the wildtype RBD (Elfiky and Ibrahim 2021a, 2022; Ibrahim et al. 2021). It appears that in the new variants, the virus increases its ability to recognize different host-cell receptors to increase its transmissibility. Therefore, we could combat the delta strain by targeting these receptors with inhibitors to reduce the probability of virus entry and vaccines that detect their binding sites on the viral spike (Elfiky et al. 2021b; Elfiky 2021; Elshemey et al. 2022).

## Conclusion

 SARS-CoV-2 delta strain is more contagious than the WT strain raising fear of the effectiveness of the current vaccination strategy. Furthermore, India reported the highest daily new infection due to this strain in May 2021. Therefore, it is essential to check for the entry mechanism of this strain, aiming to stop or decelerate the infection rate. The current study tested the potential of the main entry receptors, ACE2 and GRP78, in viral recognition. GRP78 shows the same binding affinity to RBD of the delta strain, while ACE2 affinity is slightly reduced. This congeals the effectiveness of using anti-ACE2 and anti-GRP78 as a possible route for viral fighting in the new starins of SARS-CoV-2.

## Data Availability

Data is available upon request from the corresponding author.
